# Family functioning and perceived academic achievement of adolescents from variable-centered and person-centered perspectives: the roles of teacher support and psychological capital

**DOI:** 10.3389/fpsyg.2026.1915045

**Published:** 2026-10-05

**Authors:** Lihua Wang, Yufang Zhu

**Affiliations:** 1 School of Early Childhood Education, Shangrao Preschool Education College, Shangrao, China; 2 School of Education Science, Nanjing Normal University, Nanjing, China; 3 School of Rural Education Revitalization, Shangrao Preschool Education College, Shangrao, China

**Keywords:** adolescents, family functioning, latent profile analysis, moderated mediation, perceived academic achievement, psychological capital, teacher support

## Abstract

**Introduction:**

Academic achievement is of paramount importance for adolescent development. However, how family functioning affects academic achievement through psychological capital (PsyCap), and the role of teacher support in this process, remain largely unexplored. Moreover, whether PsyCap exhibits heterogeneous profiles and how different environmental factors distinguish among them warrant further investigation.

**Method:**

A survey was conducted among 1,288 students from eight junior high schools in Jiangxi Province, China, using cluster and convenience sampling. Hayes’ PROCESS macro was used to test the moderated mediation model. Latent profile analysis (LPA) was conducted to explore the heterogeneous latent taxonomy of PsyCap.

**Results:**

Significant positive correlations were observed among family functioning, PsyCap, teacher support, and perceived academic achievement.PsyCap fully mediated the relationship between family functioning and perceived academic achievement. Teacher support significantly moderated the effect of family functioning on PsyCap and the entire mediating pathway. LPA identified three distinct PsyCap profiles: low (15%), moderate (60%), and high (25%). These profiles showed a stepwise pattern of significant differences in perceived academic achievement. The higher the levels of family functioning and teacher support, the more likely students were to be classified into the moderate or high PsyCap profiles, and teacher support exhibited a stronger effect.

**Discussion:**

By integrating variable-centered and person-centered approaches, this study reveals that family functioning indirectly promotes perceived academic achievement by cultivating PsyCap, and that teacher support strengthens this transformative process, highlighting the synergistic effects of the family and school microsystems.

## 1 Introduction

For adolescents, academic learning constitutes a pivotal developmental task (Ma et al., 2018). Their academic success not only determines their access to higher education but also profoundly influences their academic attainment during tertiary education. Academic achievement is regarded as a strong predictor of individuals’ job performance and substantially affects their prospects of obtaining desirable, challenging, and well-rewarded career opportunities in the future (Van Iddekinge et al., 2024; Ting et al., 2024).

Adolescents’ academic achievement also has significant implications for their mental health and behavioral development. Low academic achievement may trigger frustration and academic disaffection, thereby elevating the risk of internalizing and externalizing behavioral problems (Chai and Han, 2023). Meta-analytic evidence has confirmed a stable correlation between low academic achievement and violent delinquent behavior (Savage et al., 2017). By contrast, students with high academic achievement report lower levels of psychological distress and fewer aggressive behaviors (Granvik Saminathen et al., 2021).

However, many adolescents fail to achieve the expected academic outcomes. Data from OECD member countries indicate that almost a quarter (25.3%) of 15-year-olds did not reach the basic standard in reading or mathematics (OECD, 2019). Hence, investigating the determinants of adolescent academic achievement is especially essential. What shapes students’ academic performance? This issue has consistently been a central concern of educational research and practice.

In this study, academic achievement was operationalized as “perceived academic achievement,” measured through students’ self-report assessments across core subjects. Prior research has demonstrated that subjectively reported academic achievement is strongly correlated with objective examination scores and can effectively differentiate between academically high-performing and low-performing student groups (Sticca et al., 2017). Meta-analysis and recent research indicate that self-reported academic performance has been widely used among student populations (Madigan and Curran, 2021; Xu and Ma, 2026). Furthermore, given the practical constraints in obtaining standardized achievement records from participating schools, a validated self-report scale offered a feasible and consistent alternative.

According to Bronfenbrenner’s bioecological model, individual development is nested within multiple environmental systems (Bronfenbrenner and Morris, 2006). Family and school represent the two innermost and most immediate microsystems for adolescents, and their functioning exerts a foundational influence on academic development. These microsystems do not operate in isolation; rather, their synergistic interaction constitutes what is termed the mesosystem (Skinner et al., 2022). Beyond the interaction of environmental systems, students’ own positive psychological resources—namely, PsyCap—may serve as a key psychological mechanism through which mesosystemic interactions are translated into academic performance. PsyCap encompasses four dimensions—hope, self-efficacy, resilience, and optimism—that represent a set of malleable and measurable positive psychological characteristics (Luthans et al., 2007).

However, most existing studies examine the effects of family and school factors on academic achievement in isolation. Few scholars have integrated family functioning, teacher support, and PsyCap into a unified analytical framework to systematically investigate their complex interactions and mediating mechanisms. This research gap restricts a comprehensive understanding of the mechanisms underlying academic achievement and hinders the development of theoretical guidance for collaborative family - school educational practices.

According to Self-Determination Theory (SDT), when the social environment satisfies an individual’s three fundamental psychological needs-autonomy, competence, and relatedness-it triggers intrinsic motivation and promotes the internalization of extrinsic motivation, which in turn facilitates positive developmental outcomes (Ryan and Deci, 2000). Meeting adolescents’ core psychological needs reliably forecasts elevated PsyCap (Schönfeld and Mesurado, 2025). Moreover, family functioning and teacher support constitute critical environmental conditions for meeting these needs, which may further promote academic achievement by augmenting PsyCap.

Conservation of Resources Theory (COR) offers additional theoretical support for the mediating role of PsyCap. This theory contends that individuals possess an innate tendency to acquire, retain, and protect resources (Hobfoll, 2002). External environmental resources, such as positive family functioning and adequate teacher support, facilitate the accumulation of internal psychological resources, exemplified by PsyCap, which subsequently promotes positive developmental outcomes.

The Broaden-and-Build Theory (BBT) posits that positive emotions enhance people’s cognitive functioning (Fredrickson, 2001). When students exhibit high levels of PsyCap, their cognitive and behavioral repertoires expand, enabling them to address academic challenges more proactively.

These four theories provide complementary analytical perspectives for the present study. The bioecological model, at the macro level, delineates the focus of this research as the mesosystem interaction between family and school. SDT, at the micro-motivational level, elucidates how support from family and teachers enhances students’ PsyCap by satisfying their basic psychological needs. COR theory further conceptualizes PsyCap as a critical coping resource, while BBT explains how this positive psychological resource ultimately promotes academic achievement through cognitive broadening effects. Interlocked with one another, these four theories collectively form the theoretical cornerstone of this study.

### 1.1 Family functioning and academic achievement

Family functioning reflects how well family members can maintain close emotional ties, perform their designated responsibilities, address family conflicts, and communicate with one another (Zhang, 2018). It serves as a comprehensive indicator of the family system’s operational status. According to the bioecological model (Bronfenbrenner and Morris, 2006), the family is the innermost microsystem, and its functioning provides a foundational basis for children’s psychological resources and learning behaviors.

Building on this foundational role, the quality of family functioning directly influences students’ psychological development, learning behaviors, and academic performance. The family represents a crucial environmental factor affecting individuals’ academic attainment. Families characterized by a supportive atmosphere and those capable of providing emotional and psychological support can promote students’ academic performance (Delos Santos et al., 2025; Rahang and Saikia, 2026). A significant positive relationship exists between effective parent–child dialogue and children’s academic success (Du and Chiang, 2026). Family function is positively and closely associated with adolescents’ academic achievement (Alammar et al., 2022). Family functioning exerts a negative influence on problem behaviors among left-behind adolescents (Gao et al., 2025). Parental autonomy support and parent–child cohesion can promote adolescents’ academic performance (Zhang Y. et al., 2024).

### 1.2 The mediating role of PsyCap

Ecological Systems Theory holds that external factors generally exert their effects through internal factors (Brofenbrenner, 1977). PsyCap is a positive individual resource, and a high level of PsyCap serves as an important prerequisite for achieving favorable academic outcomes (Chaffin et al., 2023; Ng et al., 2024). For students facing health impairments, PsyCap serves as a buffer against the detrimental influence of school-related stress, thereby protecting academic achievement and reducing behavioral problems (Muluneh and Bejji, 2024). In general, adolescents with high PsyCap tend to have higher levels of learning engagement (Xie et al., 2022; Paloș et al., 2023). Learning engagement positively influences adolescents’ academic achievement (Abid et al., 2022; Luo et al., 2025).

Meanwhile, prior research has demonstrated that family functioning exerts a significant positive influence on middle school students’ PsyCap(He et al., 2025; Xue et al., 2023). Additionally, family functioning can significantly influence self-efficacy, hope, and resilience—the core dimensions of PsyCap (Yang et al., 2025; Zarei and Fooladvand, 2022; Peng et al., 2023; Feng et al., 2025). Furthermore, family intimacy also shapes adolescents’ psychological and behavioral outcomes(Haggerty et al., 2022). A warm and well-functioning family environment may promote individuals’ PsyCap (Xu et al., 2022). Accordingly, PsyCap may act as a mediator through which family functioning influences perceived academic achievement among adolescents.

### 1.3 The moderating role of teacher support

School is the social environment most closely connected with students’ study and daily life. When children enter school, teachers become a vital source of social support. By being well acquainted with students’ learning status, teachers can guide them in adopting appropriate learning strategies and help them overcome academic difficulties. Teacher support mainly refers to students’ perception that teachers trust and treat every student fairly, respect their opinions and feelings, and show concern for their academic performance (Sakiz et al., 2012).

Teacher support is a pivotal school resource. It drives academic achievement by fostering students’ motivation, learning engagement, academic self-efficacy, and school attachment (Affuso et al., 2023; Huang and Wang, 2023; Torbergsen et al., 2023; Han et al., 2025). Perceived teacher support markedly boosts students’ PsyCap (Yang and Lian, 2025; Chu, 2024).

From the perspective of the bioecological model, the quality of interaction between family and school depends essentially on whether they establish synergistic mesosystemic connections (Bronfenbrenner and Morris, 2006). When both family functioning and teacher support are at elevated levels, the two microsystems are better able to form a concerted force, thereby positively influencing individual development (Wu, 2024). This macro-level framework suggests that teacher support may function not merely as an independent predictor of PsyCap, but as an important boundary condition that moderates the effects of family functioning.

SDT further elucidates, from a micro-motivational perspective, why such mesosystemic interactions manifest as moderating effects. Both teacher support and family functioning play a central role in satisfying students’ three basic psychological needs (Ryan and Deci, 2000). When the environment meets the needs, individuals’ intrinsic motivation can be effectively stimulated, thereby promoting their behavioral development (Zhang et al., 2010). However, the need satisfaction derived from these two sources is not merely additive. When teacher support is high, the psychological need satisfaction elicited by family functioning can be further reinforced by the school system, thereby significantly amplifying its positive effects on PsyCap. Conversely, when teacher support is low, the need satisfaction fostered by family functioning may fail to be echoed by the school environment, and thus cannot be fully transformed into internal psychological resources. Accordingly, the present study proposes the hypothesis that teacher support plays a positive moderating role in the relationship between family functioning and adolescents’ PsyCap.

### 1.4 Person-centered research on PsyCap

The variable-centered method assumes that PsyCap is homogeneous across a sample. However, individuals may differ substantially in the configuration of their multiple dimensions. Using a person-centered statistical framework, LPA identifies profiles by grouping individuals with analogous latent traits based on composite patterns of multiple observed indicators, thereby uncovering within-group heterogeneity (Spurk et al., 2020).

LPA studies have clearly identified distinct PsyCap profiles across populations. Among Chinese college students, three profiles emerged: low self-efficacy, moderate PsyCap, and high PsyCap (Liu et al., 2026). Clinical nurse samples were categorized into four profiles: poor, moderate, well-off, and affluent (Teng et al., 2024). Junior nurses were classified definitively into low- and high-level profiles (Zhang X. et al., 2024). These findings affirm that PsyCap heterogeneity is pervasive, even as specific profile typologies differ across samples.

Integrating LPA into PsyCap research decisively addresses critical questions beyond the scope of the variable-centered approach: What subgroup differences are obscured by the average mediating effect of PsyCap? How do students with distinct PsyCap profiles differ in academic performance? Do family functioning and teacher support exert differential effects on students with varying PsyCap profiles?

### 1.5 Research gaps and research objectives

Existing studies have explored links among family functioning, teacher support, PsyCap, and academic achievement. Yet several research gaps persist.

First, research on PsyCap’s mediating mechanism lacks integration across multiple microsystems. Most studies confirm that family functioning benefits PsyCap and academic achievement, but few examine how PsyCap mediates between family functioning and achievement. It is unclear how family and teacher support together influence achievement through PsyCap, or if teacher support moderates the path from family functioning to PsyCap.

Second, most studies use the variable-centered approach and ignore the heterogeneity of PsyCap configurations. This paradigm fails to capture heterogeneous PsyCap combinations across individuals. Some research uses LPA to identify PsyCap profiles, but most LPA studies focus only on profile identification and intergroup comparison. Few studies include PsyCap profiles in theoretical models as mediators or moderators, thus missing the benefits of combining person-centered and variable-centered approaches.

Third, integrated research on adolescents is lacking. These students are in early adolescence, experiencing rapid psychological changes, increased autonomy and support needs, and higher academic pressure. The links between family functioning, teacher support, PsyCap, and academic achievement may be unique for this group. Systematic research on this population is limited.

To remedy the limitations in prior research, this study adopts a combination of variable-centered and person-centered frameworks. First, we build a moderated mediation framework: family functioning is the antecedent, perceived academic achievement the consequence, PsyCap the intermediary, and teacher support moderates the family functioning-to-PsyCap path.

Second, it uses LPA to identify PsyCap profiles among adolescents, compare perceived academic achievement across profiles, and examine how family functioning and teacher support impact each profile.

This integrated framework addresses the following research question: Does family functioning influence academic outcome through the mediating role of PsyCap? Does teacher support moderate this mediation? What latent profiles emerge among adolescents based on the four PsyCap dimensions? Are there differences in perceived academic achievement among these profiles? Do family functioning and teacher support predict perceived academic achievement differently across PsyCap profiles?

Given the above theory and literature, five hypotheses were proposed.

H1: PsyCap mediates the link between family functioning and perceived academic achievement.

H2: Teacher support moderates the association between family functioning and PsyCap.

H3: Teacher support moderates the pathway through which family functioning influences perceived academic achievement, with PsyCap as the mediator.

H4: There are distinct heterogeneous profiles of PsyCap among adolescents, and significant differences in perceived academic achievement exist across different profiles.

H5: Family functioning and teacher support differ significantly in their effects on adolescents with different PsyCap profiles.

Controlling for demographic variables such as gender, school type, and parental educational level, this study tests the above hypotheses using a sample of Chinese adolescents to provide empirical evidence for family-school collaboration in improving adolescents’ perceived academic achievement.

The innovation of this integrated framework lies in the following: the variable-centered analysis reveals what role PsyCap plays, while the person-centered analysis reveals who possesses which type of PsyCap. The combination of the two approaches can not only explain the underlying mechanism but also identify heterogeneous student groups, thereby providing empirical support for targeted and precise intervention in collaborative family and school education.

## 2 Methods

### 2.1 Participants

Cluster and convenience sampling were used. In December 2024, researchers surveyed eight junior high schools in four counties of Jiangxi Province. Based on geographic location and economic development level, one urban school and one rural school were selected from each county, yielding a total of eight schools. Within each school, one to two classes were randomly selected from each grade level (Grades 7 to 9), ensuring a balanced number of classes across urban and rural schools. In total, 30 classes were recruited. All surveys were administered on paper during regular school hours, with each class serving as the unit of administration.

In each school the survey was conducted by homeroom teachers who had received standardized training. The teachers administered the survey by first reading standardized instructions regarding confidentiality and voluntary participation. Students subsequently completed the questionnaires independently in class under teacher supervision to ensure procedural fidelity. All questionnaires were collected immediately upon completion, with the average response time ranging from 8 to 15 min.

Before sampling, school administrators, homeroom teachers, parents, and students were told about the study’s objectives, survey content, confidentiality guarantee, and voluntary participation. Informed consent forms were signed by all participants; for minors, parents or guardians signed. School administrative department approval and Institutional Review Board approval from Shangrao Preschool Education College (No. SRYZXQ20241025) were obtained.

The study distributed 1,340 questionnaires, all of which were completed and turned in. Responses missing all key variables or showing regular response patterns (e.g., selecting the same option throughout) were excluded. This process yielded 1,288 valid samples, resulting in a valid response rate of 96.1%.

Among the participants, 660 were male (51.2%), and 628 were female (48.8%). By grade level, 425 students were enrolled in Grade 7 (33%), 453 in Grade 8 (35.2%), and 410 in Grade 9 (31.8%). The age range of the participants was 11–16 years (M = 13.52). Regarding school type, 672 students were enrolled in rural schools (52.2%) and 616 in urban schools (47.8%). Turning to paternal educational attainment, 177 fathers had a primary school education or below (13.7%), 646 completed junior high school (50.2%), 338 completed senior high school (26.2%), 112 possessed a university degree (8.7%), and 15 did not report their educational background (1.2%). Similarly, for maternal educational attainment, 259 mothers had a primary school education or below (20.1%), 605 completed junior high school (47.0%), 300 completed senior high school (23.3%), 99 possessed a university degree (7.7%), and 25 did not provide relevant information (1.9%). Previous research indicates that LPA typically necessitates a minimum sample size of 500 (Meyer and Morin, 2016). The present study, comprising more than 1,200 valid participants, exceeds this requirement by a considerable margin.

### 2.2 Research instruments

Teacher support was assessed using the Other Support subscale of the Perceived Social Support Scale, originally developed by Zimet et al. (1990) and translated into Chinese by Wang et al. (1999). To better suit student participants, the label “Other Support” was revised to “Teacher Support.” The revised scale items are as follows: (1) My teacher provides me with assistance when I encounter difficulties. (2) I can share my joys and sorrows with my teacher. (3) My teacher cares about my feelings. (4) My teacher offers me support when I need it. The scale is rated from 1 (strongly disagree) to 5 (strongly agree). Averaging all item responses produced a total score, where higher scores represent more perceived teacher support. The scale attained a Cronbach’s α of 0.89 in the present research.

PsyCap was assessed using the PsyCap Questionnaire, developed by Luthans et al. (2007) and revised by Wen et al. (2009). This instrument comprises 16 items that assess four aspects: self-efficacy, optimism, hope, and resilience. Responses are recorded from 1(strongly disagree) to 6(strongly agree). We averaged the item scores; a higher average denotes stronger PsyCap. Cronbach’s α for the total scale was 0.93.

Family functioning was assessed with the Family Adaptation, Partnership, Growth, Affection, and Resolve Scale (APGAR) (Smilkstein, 1978). The scale covers the following aspects: problem solving, communication and collaboration, support for growth, emotional expressiveness, and intimacy. It is concise in content and well-suited for large-scale survey administration. The Chinese version of the scale, which has been widely employed in family functioning research, exhibits good reliability and validity (Zhou et al., 2026; Yang et al., 2026). The scale includes 5 items rated as follows: 0 (almost never), 1 (sometimes), and 2 (almost always), with the total score indicating overall functioning. Cronbach’s α was 0.81.

Perceived academic achievement was evaluated using the Academic Achievement Questionnaire (Wang et al., 2010), which includes 3 items for adolescents to self-evaluate their performance in Chinese, Mathematics, and English. Each item is rated from 1 (very poor) to 5 (excellent). We averaged the scores to represent perceived academic performance; higher average scores indicate better academic outcomes. Cronbach’s α was 0.75. Recent studies have suggested that this scale demonstrates acceptable applicability among Chinese adolescent populations (Gu et al., 2024; Hu et al., 2025).

### 2.3 Data analysis

Firstly, raw data from the paper-and-pencil questionnaires were entered and verified using EpiData 3.1. Subsequently, SPSS 25.0, the PROCESS 4.2 macro developed by Hayes, AMOS 26.0, and Mplus 8.3 were adopted for statistical analysis. The analytical procedures were conducted as follows.

First, we evaluated the reliability and common method bias of the scales. Descriptive statistics and bivariate correlations were conducted for all variables to preliminarily explore bivariate associations.

Second, a Confirmatory Factor Analysis (CFA) was conducted in AMOS 26.0 for all latent variables to assess the reliability and construct validity.

Third, the PROCESS macro (Model 4) was used to test the simple mediating effect, and the PROCESS macro (Model 7) was further employed to examine the moderated mediation effect.

Fourth, an LPA was conducted on PsyCap scores using Mplus 8.3. The number of latent classes was determined by integrating multiple fit indices, including the Akaike Information Criterion (AIC), Bayesian Information Criterion (BIC), sample-size-adjusted BIC (aBIC), Entropy, Lo–Mendell–Rubin likelihood ratio test (LMRT), and bootstrap likelihood ratio test (BLRT), so as to evaluate the classification quality (Peugh and Fan, 2013). Lower AIC, BIC, and aBIC values denote a better fit. Entropy > 0.80 means classification accuracy surpasses 90%, and values closer to 1 reflect more reliable classification (Lubke and Muthen, 2007). Significant LMRT and BLRT results (*p* < 0.05) imply that the K-class model is preferable to the (K − 1)-class model (Jung and Wickrama, 2008). Subsequently, the BCH method was adopted to examine the effect of PsyCap profiles on perceived academic achievement. Finally, the R3STEP approach was used to assess the influence of family functioning, teacher support, and demographic variables on membership in PsyCap latent profiles.

### 2.4 Reliability and validity analysis

Given that all scales employed in this study have been previously validated among Chinese adolescent populations and demonstrated good internal consistency reliability in the present sample, we did not conduct exploratory factor analysis. Instead, we directly assessed the construct validity, composite reliability, and discriminant validity of the scales through confirmatory factor analysis (CFA).

As shown in Table 1, the composite reliability (CR) values ranged from 0.76 to 0.93, all above 0.70, thereby confirming acceptable internal consistency. The average variance extracted (AVE) values ranged from 0.46 to 0.67, suggesting acceptable convergent validity for all scales.

**Table 1:** Construct reliability and validity measures.

Variables	CR	AVE	MSV	1	2	3	4
1 Family functioning	0.81	0.47	0.30	0.68			
2 PsyCap	0.93	0.46	0.34	0.55***	0.68		
3 Teacher support	0.89	0.67	0.34	0.46***	0.59***	0.82	
4 Perceived academic achievement	0.76	0.51	0.26	0.31***	0.51***	0.23***	0.72

CR, Construct Reliability, AVE Average Variance Extracted, MSV Maximum Shared Variance; and the diagonal elements represent the square roots of AVE, ***p < 0.001.

Furthermore, as presented in Table 1, each latent variable’s AVE surpassed the absolute value of its row-column correlation coefficients, demonstrating adequate discriminant validity among all latent variables.

All HTMT values were below 0.85 (Henseler et al., 2015; see Table 2), indicating sufficient discriminant validity for the variables.

**Table 2:** HTMT ratio matrix.

Variables	1	2	3	4
1 Family functioning	—			
2 PsyCap	0.54	—		
3 Teacher support	0.46	0.58	—	
4 Perceived academic achievement	0.31	0.50	0.23	—

The measurement model involving four latent variables, namely family functioning, teacher support, PsyCap, and perceived academic achievement, was tested. The model fit indices were as follows:χ^2^/df = 4.77, RMSEA = 0.05, SRMR = 0.04, CFI = 0.93, NFI = 0.92, TLI = 0.93, and IFI = 0.93. These fit indices suggest that the measurement model fits the empirical data adequately.

## 3 Results

### 3.1 Common method bias test

To mitigate multicollinearity, the independent variable, the moderator, and their interaction term were mean-centered. All models yielded VIF values between 1.01 and 1.86, far below the 5 threshold, indicating no serious multicollinearity problem in this research. Harman’s single-factor test showed that the first factor accounted for 32.27% of the variance, a value lower than 40.00%, indicating no common-method bias in the present data.

### 3.2 Correlation analysis among variables

Table 3 shows that school type was significantly positively correlated with PsyCap, teacher support, and perceived academic achievement (*p* < 0.05). Gender was significantly negatively correlated with PsyCap (*p* < 0.01). Age showed significant negative correlations with family functioning, PsyCap, teacher support, and perceived academic achievement (*p* < 0.01). Both paternal and maternal educational levels were significantly positively correlated with family functioning, PsyCap, teacher support, and perceived academic achievement (all *p* < 0.01).

**Table 3:** Descriptive statistics and the correlation (n = 1,288).

Variables	M ± SD	1	2	3	4	5	6	7	8	9
1ST	0.48 ± 0.50	1								
2gender	0.49 ± 0.50	−0.05	1							
3 age	13.52 ± 0.95	−0.11***	0.01	1						
4 PE	2.30 ± 0.82	0.32***	0.01	−0.14***	1					
5 ME	2.19 ± 0.85	0.33***	0.03	−0.10**	0.66***	1				
6 FF	5.53 ± 2.64	0.02	−0.03	−0.09**	0.10***	0.08**	1			
7 PsyCap	3.82 ± 0.98	0.12***	−0.08**	−0.09**	0.17***	0.16***	0.47***	1		
8 TS	3.09 ± 1.05	0.07*	−0.04	−0.18***	0.10**	0.09**	0.39***	0.53***	1	
9 PAA	2.58 ± 0.94	0.18***	0.02	−0.08**	0.22***	0.18***	0.24***	0.42***	0.19***	1

*P < 0.05, **P < 0.01, ***P < 0.001. ST, school type; PE, Paternal education; ME, maternal education; FF, family functioning; TS, teacher support; PAA, perceived academic achievement.

ST:0 = rural junior high schools, 1 = urban junior high chools. Gender:0 = male, 1 = female. Paternal (Maternal) education:1 = primary school, 2 = junior high school, 3 = senior high school, 4 = college or university. The same applies to the following table.

Additionally, significant positive correlations were found among family functioning, PsyCap, teacher support, and perceived academic achievement (*p* < 0.001). Because school type, gender, age, and parental education were significantly associated with either PsyCap or perceived academic achievement, we included them as controls in subsequent analyses.

### 3.3 Mediation effect analysis

All variables were first standardized. Then, we used Model 4 in SPSS PROCESS to examine whether PsyCap mediated the link between family functioning and perceived academic achievement. After controlling for school type, gender, age, paternal educational level, and maternal educational level, family functioning significantly positively influenced PsyCap (β = 0.46, t = 18.66, *p* < 0.001). PsyCap, in turn, significantly positively influenced perceived academic achievement (β = 0.36, t = 12.44, *p* < 0.001). However, the direct influence of family functioning on perceived academic achievement was non-significant(β = 0.06, t = −1.95, *p* > 0.05). Thus, PsyCap served as a complete mediator in the link between family functioning and perceived academic achievement(see Table 4 for details).

**Table 4:** The mediating effect of PsyCap.

Independent variables	Model 1: PsyCap				Model 2: PAA			
	β	SE	t	95%CL	β	SE	t	95%CL
School Type	0.14	0.05	2.70**	[0.04,0.24]	0.17	0.05	3.10**	[0.06,0.27]
Gender	−0.14	0.05	−2.85**	[−0.24,-0.04]	0.11	0.05	2.12*	[0.01,0.21]
Age	−0.02	0.03	−0.88	[−0.07,0.03]	−0.01	0.03	−0.48	[−0.07,0.04]
Paternal education	0.06	0.04	1.50	[−0.02,0.14]	0.14	0.04	3.44***	[0.06,0.23]
Maternal education	0.08	0.04	2.14*	[0.01,0.16]	0.02	0.04	0.39	[−0.06,0.10]
Family functioning	0.46	0.02	18.66***	[0.41,0.51]	0.06	0.03	1.95	[−0.01,0.11]
PsyCap					0.36	0.03	12.44***	[0.31,0.42]
R^2^	0.26				0.21			
F	70.81***				47.12***			

The Bootstrap results indicated that PsyCap significantly mediated the relationship between family functioning and perceived academic achievement among adolescents (effect size = 0.17, 95% CI [0.14, 0.21]), with the interval not including zero, confirming statistical significance.

Further calculations showed that PsyCap’s mediating effect accounted for 74.9% of the overall effect linking family functioning to perceived academic outcomes. Collectively, the results fully supported Hypothesis 1, namely that PsyCap significantly mediates the association between family functioning and perceived academic achievement among adolescents.

### 3.4 Moderated mediation effect analysis

After controlling for school type, gender, age, paternal educational level, and maternal educational level, we employed Model 7 in SPSS PROCESS to examine whether teacher support moderated the mediating pathway involving PsyCap.

Table 5 shows that Model 1 (*F* = 98.26, *p* < 0.001) and Model 2 (*F* = 47.12, *p* < 0.001) were significant overall. Family functioning (β = 0.31, *p* < 0.001) and teacher support (β = 0.40, *p* < 0.001) significantly positively affected PsyCap. PsyCap had a significant positive effect on perceived academic achievement (β = 0.36, *p* < 0.001). The interaction term of family functioning and teacher support also significantly affected PsyCap (β = 0.05, *p* < 0.05), indicating that teacher support moderated the association between family functioning and PsyCap. These findings supported Hypothesis 2.

**Table 5:** Analysis of moderated mediation.

Independent variables	Model 1: PsyCap				Model 2: PAA			
	*β*	*SE*	*t*	*95%CL*	*β*	*SE*	*t*	*95%CL*
School type	0.12	0.05	2.52*	[0.03,0.21]	0.17	0.05	3.10**	[0.06,0.27]
Gender	−0.11	0.04	−2.39*	[−0.19,-0.02]	0.11	0.05	2.12*	[0.01,0.21]
Age	0.04	0.02	1.49	[−0.01,0.08]	−0.01	0.03	−0.48	[−0.07,0.04]
Paternal education	0.07	0.04	1.54	[−0.02,0.13]	0.14	0.04	3.44***	[0.06,0.23]
Maternal education	0.07	0.04	1.86	[−0.01,0.14]	0.02	0.04	0.39	[−0.06,0.10]
Family functioning	0.31	0.02	12.88***	[0.26,0.36]	0.06	0.03	1.95	[−0.01,0.11]
Teacher support	0.40	0.02	16.19***	[0.35,0.44]				
Family functioning × teacher support	0.05	0.02	2.43*	[0.01,0.09]				
PsyCap					0.36	0.03	12.44***	[0.31,0.42]
*R^2^*	0.39				0.21			
*F*	98.26***				47.12***			

A further test of the moderated mediation effect was conducted. At low, moderate, and high levels of teacher support, the indirect effects of family functioning on academic achievement via PsyCap were 0.09, 0.11, and 0.13, respectively, and all 95% bootstrap confidence intervals excluded zero. The value of the moderated mediation index was 0.02 (bootstrapped SE = 0.01), and the 95% confidence interval [0.01, 0.04] failed to encompass zero.

The above results supported Hypothesis 3. That is, teacher support moderated the indirect path from family functioning to perceived academic achievement via PsyCap, and the indirect effect became significantly stronger as teacher support increased.

To further examine the moderating effect, teacher support was grouped into high (M + 1 SD) and low (M − 1 SD) categories based on ±1 SD from the mean, and simple slopes were then estimated (Dearing and Hamilton, 2006).

The results showed that for adolescents with low teacher support, PsyCap increased significantly as family functioning improved (β = 0.26, t = 7.73, *p* < 0.001). For students with high teacher support, the increase in PsyCap with rising family functioning was even stronger (β = 0.37, t = 10.99, *p* < 0.001). That is, teacher support moderated the influence of family functioning on adolescents’ PsyCap, enhancing its boosting effect.

Figure 1 clearly illustrates this moderating pattern. In addition, the figure shows that, at the same level of family functioning, students who perceive higher teacher support tend to have higher PsyCap.

**Figure 1:**
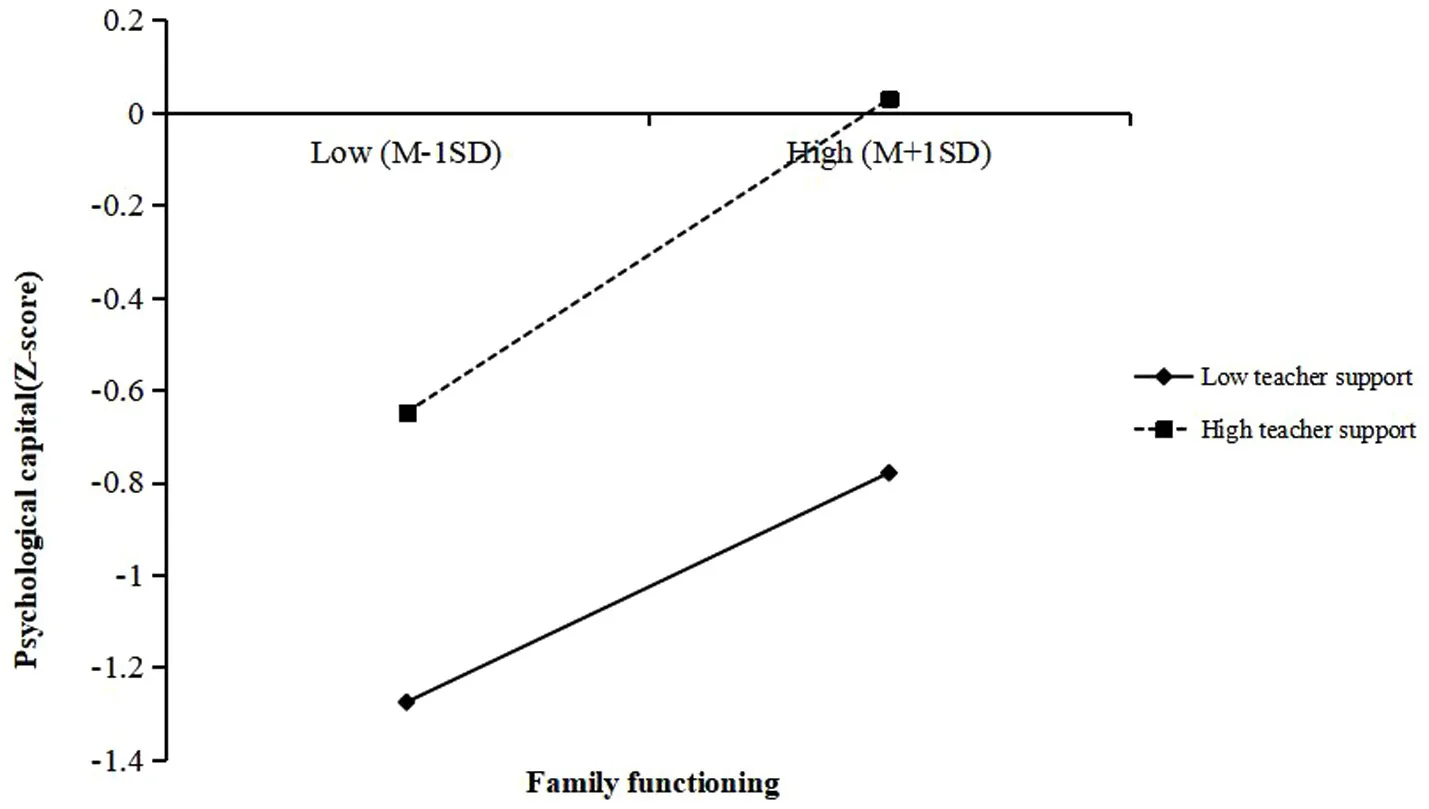
The moderating effect of teacher support.

### 3.5 LPA of PsyCap

A total of five latent profile models were fitted in the present study. The three fit indices(AIC, BIC, and aBIC)exhibited a gradual decreasing trend as the number of profiles increased. When the number of latent classes was set to 3, the Entropy value reached its peak, and both LMRT and BLRT yielded statistically significant differences (*p* < 0.001).

The LMRT results indicated no statistically significant difference when the number of classes was 4 (*p* = 0.122). A five-class model yielded a smallest latent class of only 4%, a size too small to ensure dependable interpretability. After a comprehensive evaluation of model fit, Model 3 was selected as the optimal latent class solution (see Table 6). Based on Model 3 and the PsyCap indicators, a latent profile plot was constructed to visually illustrate the differences among the latent classes (see Figure 2).

**Table 6:** Fit indices for latent profile models of PsyCap (*n* = 1,288).

Profile	AIC	BIC	aBIC	Entropy	LMRT	BLRT	Proportion (%)
1-profile	16057.65	16098.94	16073.53	–	–	–	100
2-profile	14563.09	14630.18	14588.88	0.75	<0.001	<0.001	54/46
3-profile	13736.83	13829.73	13772.55	0.84	<0.001	<0.001	15/60/ 25
4-profile	13495.39	13614.09	13541.03	0.79	0.122	<0.001	10/41/38/11
5-profile	13366.33	13510.83	13421.89	0.80	0.002	<0.001	4/16/45/28/7

**Figure 2:**
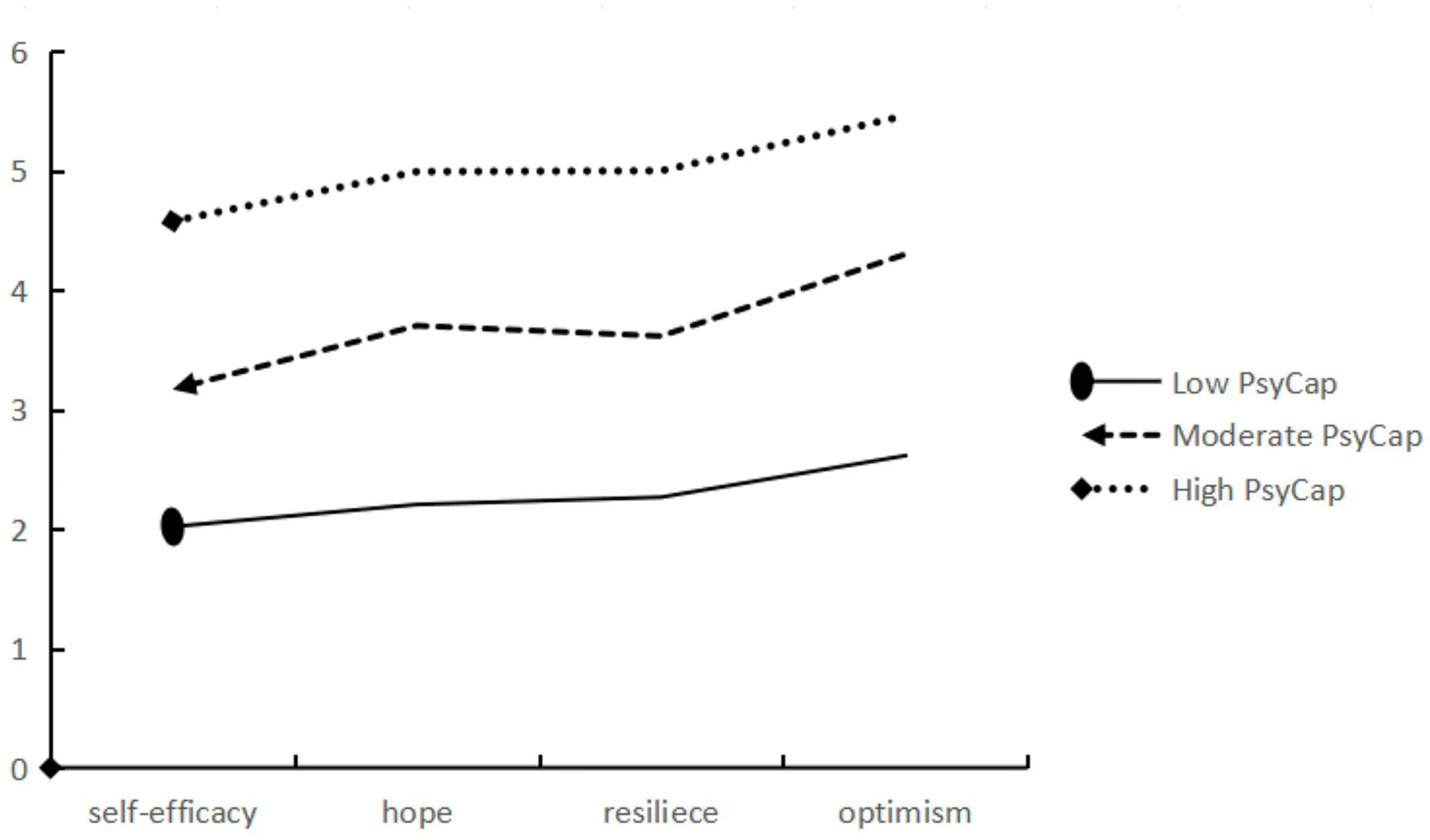
Mean scores on the four PsyCap dimensions by latent profile.

Based on each dimension’s scores, the profiles were divided into three categories: C1, C2, and C3. Category 1, representing 15% of adolescents with universally the lowest scores, was designated as the low-level PsyCap profile. Category 2 comprised 60% of adolescents, whose scores in each dimension were higher than those of Category 1 but lower than those of Category 3, and was named the moderate-level PsyCap group. Category 3 comprised 25% of adolescents and recorded the highest scores on all dimensions, representing a high-level PsyCap group.

The modified BCH technique (Mplus 8.3) was adopted to examine significant inter-profile differences in perceived academic achievement among adolescents exhibiting heterogeneous PsyCap profiles. As shown in Table 7, PsyCap profile membership was associated with a significant difference in perceived academic achievement (*F* = 236.34, *p* < 0.001, Partialη^2^ = 0.18). Post-hoc pairwise comparisons further revealed that students in the high PsyCap group scored significantly higher on perceived academic achievement than those in the moderate PsyCap group, who, in turn, outperformed those in the low PsyCap group. These findings fully supported Hypothesis 4.

**Table 7:** Differences in perceived academic achievement among three PsyCaps.

Variables	N (%)	M ± SD	Statistic (*p* value)	*Post-hoc* test	Partial η^2^
Low PsyCap ①	196 (15)	1.91 ± 0.06	236.34(p<0.001)	①<②<③ (p<0.001)	0.18
Medium PsyCap ②	768 (60)	2.51 ± 0.04			
High PsyCap ③	324 (25)	3.14 ± 0.05			

We set PsyCap’s latent profile membership as the outcome variable, with school type, gender, age, parental education, family functioning, and teacher support as predictors. With the low PsyCap profile serving as the reference group, we performed a logistic regression analysis with the R3STEP procedure in Mplus 8.3.

Results (see Table 8) revealed that, in terms of demographic variables, urban students were significantly more prone to fall into the moderate PsyCap category(OR = 1.74, 95%CI = [1.15, 2.64], *p* = 0.009) and the high PsyCap profile (OR = 1.79, 95%CI = [1.17, 2.74], *p* = 0.008) compared to rural students. Higher maternal educational level was significantly associated with an increased likelihood of being assigned to the high PsyCap profile (OR = 1.58, 95% CI = [1.16, 2.14], *p* = 0.003). Conversely, older students had a significantly lower probability of belonging to the high PsyCap profile (OR = 0.80, 95% CI = [0.65, 0.99], *p* = 0.049). Importantly, gender and paternal educational level did not demonstrate a significant effect in latent profile membership in the present analysis.

**Table 8:** Multiple logistic regression of antecedents on PsyCap profile categories.

Variables	Profile 2 (vslowPsyCapgroup)				Profile 3 (vslowPsyCapgroup)			
	*β*	*OR*	95%CI	*P*	*β*	*OR*	95%CI	*P*
School type (“rural school” as ref)	0.56	1.74	[1.15, 2.64]	0.009	0.58	1.79	[1.17, 2.74]	0.008
Gender (“male” as ref)	0.14	1.16	[0.79, 1.69]	0.460	−0.40	0.67	[0.44, 1.01]	0.056
Age	0.06	1.06	[0.86, 1.30]	0.596	−0.22	0.80	[0.65, 0.99]	0.049
Paternal education	0.22	1.25	[0.90, 1.74]	0.182	0.29	1.34	[0.96, 1.88]	0.090 0.003
Maternal education	0.26	1.29	[0.96, 1.74]	0.087	0.46	1.58	[1.16, 2.14]	
Family functioning	0.26	1.30	[1.18, 1.43]	<0.001	0.51	1.67	[1.48, 1.89]	<0.001
Teacher support	0.61	1.83	[1.47, 2.29]	<0.001	1.64	5.15	[3.73, 7.12]	<0.001

OR, Odds ratio; CI, Confidence interval.

With respect to family functioning, relative to the low PsyCap profile, higher family functioning scores were significantly linked to a greater probability of being categorized into both the moderate PsyCap profile (OR = 1.30, 95% CI = [1.18, 1.43], *p* < 0.001) and the high PsyCap profile (OR = 1.67, 95% CI = [1.48, 1.89], *p* < 0.001). Similarly, higher teacher support significantly enhanced the likelihood of membership in the moderate PsyCap profile (OR = 1.83, 95% CI = [1.47, 2.29], *p* < 0.001) and the high PsyCap profile (OR = 5.15, 95% CI = [3.73, 7.12], *p* < 0 0.001). Empirically, these results corroborate Hypothesis 5.

## 4 Discussion

### 4.1 The mediating role of PsyCap

The present study found that the direct influence of family function on perceived academic achievement became non-significant after controlling for PsyCap, whereas the indirect effect remained significant. This indicates that PsyCap exerted a full mediating effect.

The present findings suggest that family influence on academic outcomes is indirect, operating through the activation of students’ internal psychological resources. In other words, well-functioning families provide fertile ground for the development of PsyCap, which in turn serves as the direct driving force behind academic achievement. This discovery offers a novel theoretical perspective for understanding the relationship between family environment and students’ academic performance. Evidence to date suggests that this study pioneers the examination of PsyCap as a complete mediator in the association between family functioning and adolescents’ perceptions of their academic success. This raises the question: which specific family dynamics activate these internal resources? Below, we provide a unified explanation from three theoretical perspectives—SDT, COR theory, and BBT.

From the perspective of SDT, the core feature of well-functioning families lies in their capacity to satisfy students’ three basic psychological needs (Ryan and Deci, 2000). Specifically, when family members maintain close emotional bonds, students’ need for relatedness is satisfied; when parents respect their children’s autonomous choices and provide reasonable academic expectations, students’ needs for autonomy and competence are, respectively, supported. Fulfilling these essential psychological needs may be associated with adolescents’ PsyCap, thereby advancing their academic engagement and scholastic success (Carmona-Halty et al., 2019). Studies focusing on high school and college students have demonstrated that adolescents with positive parent–child relationships and sufficient parental support maintain higher academic PsyCap, thereby achieving better academic outcomes (Carmona-Halty et al., 2022; Li et al., 2022).

COR theory further elucidates how this psychological state, activated by family functioning, can be stably transformed into a lasting psychological resource. When families consistently provide a supportive environment, students receive a safe resource reservoir (Hobfoll et al., 2023). They not only derive positive psychological energy from family interactions but also avoid expending resources coping with conflict or emotional deprivation, allowing them to allocate more resources to exploration, learning, and academic challenges. This ongoing resource accumulation facilitates the development of internal resources such as PsyCap (Zhong et al., 2023), enabling individuals to cope with academic stress, resist psychological depletion, and achieve favorable behavioral outcomes.

BBT provides a cognitive-level explanation for understanding how PsyCap ultimately facilitates academic achievement. Activated by family functioning, PsyCap—as a positive emotional–cognitive composite state—broadens students’ scope of attention and thought-action repertoires, enabling them to become more flexible, open, and creative in the learning process, and thereby to construct more effective learning strategies and more positive academic self-concepts (Fredrickson, 2001). PsyCap has been confirmed as the strongest proximal predictor of academic engagement (Deng et al., 2026). This suggests that enhancements in PsyCap drive students’ active participation in scholastic activities, which in turn facilitates academic development.

### 4.2 The moderating effect of teacher support

The present study found that teacher support exerts a positive moderating effect on the relationship between family functioning and PsyCap. Although the effect size is relatively small, its statistical significance suggests that teacher support may influence how family functioning exerts its effects. This moderating effect can be interpreted at a macro level from the mesosystem perspective of the bioecological model. The school and the family may achieve a degree of mutual coordination in emotional support and value guidance, and such mesosystemic congruence helps facilitate the nurturing effect of family functioning on PsyCap more fully (Bronfenbrenner and Morris, 2006). The present study provides preliminary empirical evidence for this macro-level framework, suggesting that teacher support may play an auxiliary role in the process through which family functioning exerts its influence.

SDT further elucidates the psychological processes underlying the synergistic effects within this mesosystem. The core functions of both family functioning and teacher support lie in satisfying students’ three basic psychological needs (Ryan and Deci, 2000). Teachers foster autonomy by encouraging self-directed choice and fulfill the need for relatedness through emotional investment (Affuso et al., 2023), thereby cultivating an enabling educational environment that supports the development of students’ PsyCap (Sava et al., 2020). When the need satisfaction derived from teacher support converges with the emotional bonding and respect for autonomy provided by family functioning, they may generate complementary effects that amplify the enhancement of PsyCap. Recent meta-analytic evidence corroborates that the fulfillment of basic psychological needs across distinct microsystems exhibits interactive effects, collectively predicting positive developmental outcomes in individuals (Howard et al., 2021). Conversely, when teacher support is deficient, the need satisfaction fostered by family functioning may fail to fully internalize into stable psychological resources due to the lack of resonance from the school environment.

From the perspective of resource dynamics within COR theory, teacher support constitutes an external resource provided by the school microsystem. According to this framework, individuals are inherently motivated to acquire, retain, and protect resources, whereby resource loss exerts a disproportionately stronger impact than resource gain (Hobfoll et al., 2018). When teachers consistently offer assistance and emotional solicitude, these behaviors can reduce the psychological resources students must expend to navigate teacher-student relationships, thereby freeing additional resources for investment in academic development. Under such conditions, the nascent psychological resources fostered by family functioning are more likely to be conserved. Conversely, when teachers fail to exhibit such supportive behaviors, students may be compelled to mobilize supplementary psychological resources to cope with relational uncertainty or emotional detachment within the teacher-student dynamic. This process potentially undermines the resource-generating efficacy of family functioning. This resource-dynamic interpretation aligns logically with the mesosystem proposition of the bioecological model: the degree of alignment between resource provisions across microsystems may critically influence the efficiency of individual resource accumulation.

In summary, this study aims to clarify the moderating influence of teacher support on the relationship between family functioning and PsyCap through three theoretical frameworks. The mesosystem perspective offers a framework for understanding the structural context (i.e., the quality of linkages between two microsystems). SDT illuminates the underlying psychological processes (i.e., the compensatory interplay of need satisfaction). COR theory reveals potential resource mechanisms (i.e., resource conservation versus depletion). These perspectives are mutually complementary, collectively providing a macro-to-micro theoretical interpretation of this moderating effect—spanning from the broader environmental context to specific psychological mechanisms. However, given the modest effect size observed for this moderation, these theoretical explanations should be treated as hypotheses requiring further empirical validation.

### 4.3 Heterogeneous profiles of PsyCap

Another core finding of the present study is that adolescents exhibit three distinct latent profile patterns of PsyCap: low (15%), moderate (60%), and high (25%). These profiles display a stepped gradient across the four dimensions of hope, self-efficacy, resilience, and optimism (*p* < 0.001). This typological structure has been preliminarily validated among Chinese college students. Song et al. (2025) conducted a LPA with 541 Chinese undergraduates and identified three profiles: low PsyCap (38.1%), high PsyCap (50.5%), and a dominant loss-oriented profile (11.4%). Nevertheless, the proportional distribution of each profile differed substantially from that observed in the current study.

Among the three PsyCap profiles of adolescents in this study, the moderate profile accounted for the largest proportion. This suggests that most adolescents remain at a marginal yet suboptimal level of PsyCap, leaving considerable room for upward improvement. This finding also provides a valuable foundation for future educational intervention design.

Results from the modified BCH method indicated that PsyCap profile membership explained approximately 18.3% of the variance in academic achievement. Notably, the academic achievement gap between the high and low PsyCap profiles was particularly pronounced, with a mean difference of 1.228, which carries clear practical educational implications. Such a discrepancy may correspond to several percentile ranks in standardized academic examinations. This result aligns closely with the propositions of COR. Students with a high PsyCap profile possess abundant internal resources, which facilitate the formation of resource-gain spirals. That is, existing resources enable individuals to acquire further new resources, thereby establishing a virtuous cycle. In contrast, students with a low PsyCap profile may fall into resource-loss spirals, in which resource scarcity undermines their capacity to cope with academic challenges and further widens the achievement gap.

From the BBT perspective (Fredrickson, 2001), those with high PsyCap are more prone to positive emotions (e.g., hope and pride), which broaden their cognitive and behavioral repertoires, foster proactive learning engagement and deep learning approaches, and ultimately contribute to superior academic outcomes.

Regarding predictive variables, R3STEP regression results indicated that teacher support was a critical determinant of PsyCap profile membership. Each unit increase in teacher support raised the odds of high (vs. low) PsyCap profile membership by 5.15 times, an effect far exceeding that of family functioning (1.67 times). Similarly, the odds of the moderate PsyCap profile were also significantly higher for teacher support (OR = 1.83) than for family functioning (OR = 1.30). This evidence supports a central inference about how teacher support moderates: it acts as a pivotal factor enabling adolescents to transition from low to moderate and high levels of PsyCap.

The present study further indicated that urban students exhibited 74.3% and 78.7% higher odds of belonging to the moderate and high PsyCap profiles, respectively, compared with rural students (OR = 1.743 and OR = 1.787). The sample was drawn from Jiangxi Province, located in central China, which is characterized by a prototypical urban–rural dual structure. The region experiences high rates of outmigration among rural adults, resulting in a substantial proportion of left-behind adolescents and relatively scarce educational resources. Such structural constraints likely impede the effective cultivation of psychological resources by families and schools. From the lens of COR theory, these urban–rural disparities can be interpreted as structural differences in resource environments. Urban students are situated within more resource-rich ecologies, where supportive inputs from families and schools complement and accumulate synergistically, facilitating a spiral of resource gain. In contrast, rural students face the dual risks of constrained access to resources and ongoing resource depletion. These findings suggest that interventions targeting rural adolescents and their families should extend beyond individual-level psychological remediation to incorporate systemic resource compensation. Specifically, schools could provide supplemental psychological counseling and academic support to partially offset deficits in familial resources.

Students with more educated mothers were significantly more prone to belong to the high PsyCap category (OR = 1.58, *p* = 0.003). This finding can be understood more deeply against the socioeconomic backdrop of Jiangxi Province. As an inland province in central China, Jiangxi exhibits pronounced urban–rural disparities in maternal educational attainment at the household level. In urban areas, mothers generally attain higher levels of education, whereas in parts of rural Jiangxi, mothers’ years of schooling remain comparatively limited—a structural condition that directly bears upon the quality and style of family upbringing. Research indicates that more highly educated mothers tend to adopt autonomy-supportive parenting styles (Su-Russell and Russell, 2021), enabling them to provide more effective academic guidance, emotional support, and positive role modeling for their children, thereby fostering the development of positive psychological assets such as hope, self-efficacy, and optimism. In a province like Jiangxi where educational resources are unevenly distributed, maternal education serves not merely as an indicator of family socioeconomic status but as a critical proximal factor that directly shapes the cultivation of adolescent PsyCap.

Notably, paternal education failed to emerge as a significant predictor in the present study—a pattern that carries cultural plausibility within the Chinese context. Rooted in prevailing cultural norms regarding gendered labor, mothers predominantly serve as the primary caregivers responsible for children’s daily routines and academic affairs. Consequently, maternal educational philosophies and parenting practices tend to exert a more immediate and lasting impact on child psychological development. This finding yields clear implications for home–school collaborative interventions: in Jiangxi and demographically similar regions, particular attention should be directed toward families with lower maternal education. Supplemental educational support and parenting-strategy guidance—delivered through vehicles such as parent schools or mothers’ workshops—could help compensate for deficits in family-based resources.

The study also revealed that older students were significantly less likely to fall into the high PsyCap profile. This noteworthy result indicates that PsyCap among adolescents tends to decline as grade level increases from Grade 7 to Grade 9. One plausible explanation is that physical changes, intensified academic pressure, and heightened needs for autonomy may lead to fluctuations in self-efficacy and reduced optimism during early adolescence. In addition, Grade 9 students face intense pressure from the senior high school entrance examination; such a high-stakes testing context may undermine their sense of hope and resilience.

In summary, the present study conceptually integrates the synergistic effects of family and school as mesosystemic interactions and examines the mediating role of PsyCap, thereby organically combining the bioecological model with SDT, COR theory, and BBT. This not only provides new evidence for understanding the environment-individual interactive mechanisms underlying adolescents’ academic development, but also indicates that the mesosystem exerts its influence by cultivating PsyCap as an internal resource.

### 4.4 Limitations and future directions

Three key limitations should be recognized and tackled in future investigations.

First, causality cannot be determined due to the cross-sectional design. Given the design, all variables were measured at one time point. Accordingly, it is difficult to infer the causal ordering among family functioning, PsyCap, and academic achievement.

Second, relying exclusively on students’ self-reports for all variables may introduce common-method bias. Although this study employed several procedural and statistical remedies to control for common-method bias—including anonymous responses, mean-centering of variables, and Harman’s single-factor test—all data were derived from student self-reports. Consequently, common method variance stemming from social desirability and consistency motives may not be entirely ruled out.

Third, the sample’s limited representativeness limits the extent to which the findings can be generalized. The sample was recruited from only several junior high schools in Jiangxi Province, thereby mainly reflecting the characteristics of rural and county-level areas in central China. Regional and cultural differences may affect the associations between family functioning, teacher support, and PsyCap. Therefore, the present findings should be generalized cautiously.

In view of the above limitations, future research can be extended from the following three perspectives.

First, longitudinal designs or experimental interventions are recommended to establish causal evidence. Future studies may adopt multi-wave longitudinal research and conduct cross-lagged panel analysis to examine the dynamic causal relationships among family function, PsyCap, and academic achievement. Alternatively, randomized controlled trials can be designed to verify the causal effect of PsyCap interventions on academic achievement.

Second, researchers should employ multiple sources to reduce common-method bias. Future research should employ standardized test scores or teacher ratings as indicators of academic achievement, thereby enhancing the validity of the findings and enabling a more objective assessment of the relationships among variables.

Third, expand the sampling scope and conduct cross-regional and cross-cultural comparative studies. Future investigations can recruit adolescent samples from diverse geographical and cultural backgrounds to test the cross-contextual stability of the present findings.

### 4.5 Practical implications

The current results have clear practical value for educational settings and can be implemented as concrete measures from three standpoints.

First, home–school collaboration should center on cultivating PsyCap. This research shows that family functioning and teacher support operate, in part or in full, through PsyCap. These findings suggest that PsyCap serves as a critical psychological bridge linking the family and school microsystems, and acts as a potential leverage point for disrupting the constraining effects of family background on academic achievement. Even among students from backgrounds characterized by weaker family functioning, schools can effectively enhance academic outcomes through systematic PsyCap interventions. Extant intervention research substantiates the malleability of PsyCap, demonstrating that it can be significantly enhanced via structured training programs. For instance, strength-based student mentoring implemented among ninth-grade students has been shown to markedly elevate their PsyCap levels (Katajisto et al., 2021). Similarly, school-based PsyCap intervention programs contribute meaningfully to the development of adolescents’ psychosocial competencies (Finch et al., 2023).

At the family level, enhancing overall family functioning requires deliberate improvements in emotional communication, increased autonomy support, and the establishment of realistic academic expectations. At the school level, institutions can organize regular parent workshops centered on topics such as “supportive parenting practices” and “fostering hope and resilience in children,” equipping parents with concrete parenting skills. Furthermore, schools may distribute accessible guides on cultivating PsyCap, supplemented with practical parent–child interaction exercises designed for daily implementation. Such initiatives facilitate the internal discovery and sustained activation of latent familial resources, thereby reinforcing the home–school partnership in promoting student development.

Second, priority should be accorded to investing in teacher support to leverage its multiplicative effect on student PsyCap. The present study revealed that a one-unit increase in perceived teacher support corresponded to a 5.15-fold increase in the odds of students belonging to the high PsyCap group relative to the low PsyCap group. This finding indicates that, particularly in resource-constrained educational settings such as parts of rural Jiangxi Province, prioritizing investment in teacher support may yield higher returns than diffusing resources across disparate initiatives. Specifically, educational authorities and school administrations could implement systematic training across three dimensions: First, training in academic feedback skills, wherein teachers are instructed to provide specific, constructive feedback during homework grading and classroom interactions to bolster students’ sense of competence. Second, training in teacher–student relationship building, which equips teachers with active listening techniques, empathetic communication strategies, and protocols for individualized counseling sessions to fulfill students’ need for relatedness. Third, training in strengths-based engagement strategies, which encourages teachers to employ praise-oriented assessments and strength-spotting techniques in daily instruction to enhance students’ self-efficacy and optimism.

Third, implement precision-targeted, differentiated tiered interventions. This study identified three distinct PsyCap profiles and demonstrated how factors such as urban–rural locale and maternal education predict profile membership—providing robust empirical evidence for stratified intervention. Schools should leverage large-scale mental health screenings administered at the start of the academic term, combined with information gathered by homeroom teachers during home visits, to preliminarily identify students’ PsyCap levels and the status of their familial resources. Intervention intensity and strategies should then be tailored according to these profiles.

Students categorized into the low PsyCap group—who often face resource deficits in both family and school contexts—are unlikely to benefit sufficiently from single-system interventions. Consequently, a coordinated, individualized intervention plan involving the school psychologist, homeroom teacher, and parents is warranted. Such plans may include weekly individual counseling sessions, regular home visits by the homeroom teacher to strengthen home–school communication bridges, and after-school academic tutoring provided by subject teachers to supply external resources.

For the moderate PsyCap group, which constitutes most of the sample, interventions can focus on universal strategies. Examples include regular class meetings centered on a growth mindset, SMART goal-setting for goal decomposition and motivation, and group-based psychological enrichment activities. These efforts aim to facilitate a collective upward shift toward higher PsyCap levels.

Interventions for the high PsyCap group should focus on consolidation and extension. Strategies may involve training these students to serve as peer supporters for mental health within their classrooms or assigning more cognitively demanding learning tasks to further stimulate their potential.

## 5 Conclusion

Integrating ecological systems theory, SDT, COR, and BBT, we proposed a moderated mediation model. Adopting a dual perspective that combines variable-centered and person-centered approaches, the present study systematically investigated the comprehensive mechanisms underlying family functioning, teacher support, and PsyCap in adolescents’ perceived academic achievement. The main findings are summarized as follows:

(1) PsyCap fully mediated the association of family functioning with perceived academic outcomes.
(2) Teacher support significantly moderated the family functioning–PsyCap link and the overall indirect pathway. Specifically, the positive effect of family functioning on PsyCap became stronger at higher levels of teacher support; correspondingly, the indirect effect of family functioning on academic achievement via PsyCap was also amplified.
(3) Three distinct latent profiles of PsyCap were identified among adolescents: low, moderate, and high levels. Family functioning and teacher support have substantial impacts on PsyCap profile membership. Compared with those in the low and moderate profiles, elevated teacher support greatly increases the odds of students belonging to the high PsyCap profile.

The present study’s theoretical contributions lie mainly in three aspects.

First, this study organically integrates the variable-centered and person-centered paradigms to construct an integrated analytical framework from a dual perspective. The variable-centered analysis reveals PsyCap’s full mediating role between family function and perceived academic achievement, addressing the mechanistic question of PsyCap’s role. By contrast, the person-centered analysis identifies three latent PsyCap profiles among adolescents through LPA, answering the heterogeneity question of who possesses which level of PsyCap. The results further indicate that family functioning and teacher support strongly influence profile membership, with the effect size of teacher support being considerably larger than that of family functioning. This integrated framework provides an empirical foundation for targeted intervention in home–school collaborative educational practice.

Second, by integrating multiple theoretical perspectives, this study constructs and validates the mediating model of family function → PsyCap → academic achievement. This full mediation pattern reveals the underlying mechanism by which external environmental resources are transformed into internal psychological resources, which in turn promote developmental outcomes. Positive family functioning provides a nurturing context for students’ PsyCap development, whereas PsyCap serves as the direct driving force of perceived academic achievement. This finding resonates with the core proposition of an integrative multi-theoretical framework, which posits that external resources facilitate the accumulation of internal resources within individuals, subsequently translating into positive developmental outcomes.

Third, this study introduces teacher support as a moderator to examine the interactive mechanisms between the two key microsystems, family and school. We observed a significant increase in the indirect effect—from family functioning to perceived academic achievement through PsyCap—at higher levels of teacher support. This pattern provides strong support for the bioecological concept of mesosystem interactions, showing how family and school resources combine to produce synergistic effects.

## Data Availability

The raw data supporting the conclusions of this article will be made available by the authors, without undue reservation.
